# Proximate and ultimate causes of supernatural beliefs

**DOI:** 10.3389/fpsyg.2022.949131

**Published:** 2022-11-17

**Authors:** Michiel van Elk

**Affiliations:** Institute of Psychology, Leiden University, Leiden, Netherlands

**Keywords:** supernatural beliefs, cultural learning, dualism, agency detection, cross-cultural, replication, cognitive science of religion (CSR), psychology of religion and spirituality

## Introduction

In the 2000s with the discovery of the so-called *God-spot*—a brain region that was suggested to be involved in the experience of God (Biello, 2007)—the field of neurotheology came to flourish, according to which supernatural beliefs are engrained in our brain. At the same time, other researchers have pointed out the relevance of socio-cultural factors for the learning and proliferation of supernatural beliefs (Norenzayan and Gervais, 2013), in line with the view that ultimately religion evolved through a process of cultural evolution, thereby fostering in-group cohesion and cooperation (Norenzayan et al., 2016). Still others have argued that religion primarily fulfills an epistemic need to understand and predict the world (Kay et al., 2010) and that it provides a palliative mechanism to cope with the fear of death (Vail et al., 2010).

**Figure 1:**
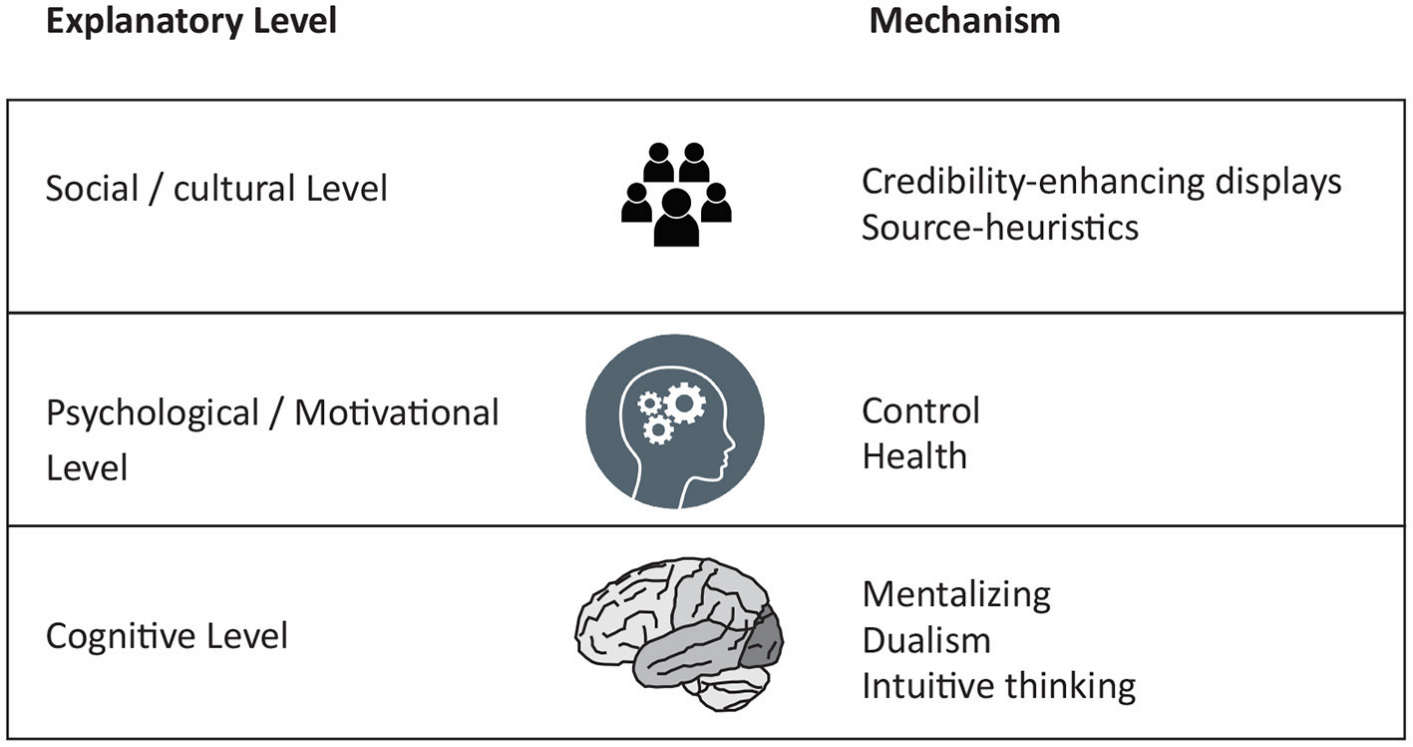
Overview of the different explanatory levels **(Left)** and the hypothesized mechanisms **(Right)** that have been proposed to account for the emergence and proliferation of supernatural beliefs.

Which of these viewpoints is right? What are the proximate and ultimate mechanisms that help us to understand why some people believe in supernatural phenomena, like an afterlife, spirit communication or a soul, whereas others don't? In this perspective paper I will provide a critical examination of the existing literature on this topic, especially in light of the so-called replication crisis: many published findings in the scientific literature turned out not to be replicable (Nosek et al., 2015). This was mainly related to questionable research practices, underpowered studies, lack of independent replication studies and the file-drawer problem and similar concerns have haunted the psychology and cognitive science of religion as well (van Elk et al., 2015; Charles et al., 2019). Therefore, in the Religious Replication Project (Hoogeveen and van Elk, 2018), over the past years we set out to assess the replicability of key findings in the field, by conducting direct replication studies of existing findings, registered report studies and large-scale cross-cultural replication studies. In this review I will specifically focus on what we learned about the (1) proximate cognitive mechanisms underlying supernatural beliefs, (2) the psychological functions subserved by supernatural beliefs, and (3) socio-cultural mechanisms contributing to the proliferation of supernatural beliefs (see Figure 1). I will end by discussing the implications of these different mechanisms for our understanding of the nature of supernatural beliefs and how they come about.

## Cognitive mechanisms underlying supernatural beliefs

Different proximate cognitive mechanisms have been proposed in the literature to underlie supernatural beliefs, including the ability to mentalize (Barrett, 2000), dualistic reasoning (Bering et al., 2005) and intuitive thinking (Boyer, 2001).

### Mentalizing

The ability to apply theory of mind reasoning has been suggested to be a necessary prerequisite for enabling belief in an anthropomorphic supernatural agent and it has been found for instance that personal prayer to God is associated with the activation of brain regions involved in mentalizing (Schjoedt et al., 2009). Hyper-mentalizing, i.e., the tendency to attribute intentions to natural phenomena such as thunderstorms and earthquakes, has also been associated with an increased tendency to believe in supernatural and paranormal phenomena (Willard and Norenzayan, 2013). By using correlational designs, across several studies we were also able to show that stronger supernatural beliefs were associated with a stronger bias for illusory agency detection by using perceptual decision making tasks in which participants were required to indicate whether a human agent was visible in a display or not (van Elk, 2013, 2015). It has also been found that mentalizing deficits, e.g., as observed in people scoring high on the autism spectrum, are negatively related to belief in a personal god (Norenzayan et al., 2012). We replicated this finding in a large-scale cross-cultural study including more than 65,000 participants, showing that an increased mentalizing ability was indeed positively associated with supernatural beliefs (Maij et al., 2017). Thus, the hypothesized relation between mentalizing and supernatural beliefs appears robust, even though reported effect sizes are small and several studies have highlighted that despite mentalizing deficits, people scoring high on the autism quotient can still endorse supernatural beliefs and have supernatural encounters (Schaap-Jonker et al., 2013; Visuri, 2020).

### Dualism

According to the naturalness of religion hypothesis (Bloom, 2007), humans have an early developing tendency to reason dualistically about the mind and the body. This tendency may be deeply engrained in our brain as we appear to have separate brain networks involved in reasoning about mental states (i.e., the theory-of-mind network and the default-mode-network) and for engaging in bodily processing (i.e., the fronto-parietal attention network; cf., Milliere, 2017). The bias for mind-body dualism already becomes prevalent from an early age onwards (Bering and Bjorklund, 2004; Bering et al., 2005): young children have a predisposition for applying dualistic reasoning about the mind and the body as being two separate entities, which might be at the basis of afterlife beliefs. In a large-scale cross-cultural study (using data collected in 24 countries across all 6 continents and including more than 10,000 participants) we set out to test the apparent cross-cultural universality of dualistic thinking (Hoogeveen and van Elk, submitted). To this end we presented participants with a vignette (using a similar design as: Giménez and Harris, 2005) describing a grandmother who passed away, and we asked participants to make continuity judgments about physical (e.g., “Do you think she can still be hungry?”) and mental states (e.g., “Do you think she can still love Bill?”). Overall, we found evidence for the hypothesis that the tendency to make continuity judgments for mental compared to physical states was cross-culturally prevalent, as participants judged mental states to be more likely to continue to exist than mental states. However, at the same time most people indicated cessation rather than continuation for all states (i.e., the modal response was to indicate that both mental and physical states would cease to exist after a person died), calling into question the apparent universality of mind-body dualism. Instead, the data appear more in line with an intuitive materialism account (Barrett et al., 2021), according to which the default is to view death in biological terms upon which all mental activity ends.

### Intuitive thinking

Dual-process accounts of religion suggest that supernatural beliefs are primarily related to an intuitive (compared to an analytical) thinking style, whereas disbelief is related to analytical thinking (Pennycook et al., 2012). In other words: believers may be more prone to accept intuitive ideas and may have a reduced tendency for detecting cognitive conflict between potentially contradictory beliefs. An initial study attempted to show that priming analytical thinking reduces supernatural beliefs (Gervais and Norenzayan, 2012), however this finding could not be replicated in a high-powered replication study (Sanchez et al., 2017). In a large-scale cross-cultural study moreover, we found that the hypothesized relationship between religiosity and intuitive thinking was cross-culturally highly variable and only became apparent in three out of the 13 countries that were included (Gervais and Norenzayan, 2018). Other research also calls into question the presumed generic relationship between conflict detection and religiosity. For instance, in a registered report fMRI study we failed to find evidence for a negative relationship between religiosity and neural conflict responses (i.e., activity in the anterior cingulate cortex in response to a Stroop-task; cf., Hoogeveen et al., 2020). Other labs have shown similar null-results when attempting to replicate the relation between religiosity and intuitive thinking (Farias et al., 2017). The lack of a consistent relationship between intuitive thinking, conflict detection and supernatural beliefs could well be related to the lack of ecologically valid measures. For instance, the cognitive reflection task—one of the most widely used measures to assess analytical thinking—has been criticized for conflating mental abilities with processes (Blacksmith et al., 2019) and it is questionable whether making errors on a Stroop task relates in any meaningful way to the anxiety-relieving effects of religion.

In sum, there appears to be mixed evidence for the role of mentalizing, dualistic reasoning and intuitive thinking as cognitive precursors underlying supernatural beliefs.

## Psychological functions subserved by supernatural beliefs

It has often been suggested that religion and supernatural beliefs can provide a palliative mechanism for coping with stressful events (Inzlicht et al., 2011), resonating with Karl Marx's adage that religion is opium for the people. Specifically, it has been suggested that religion helps us to cope with a lack of control and can provide direct benefits for one's mental and physical health.

### Control

According to compensatory control theory (CCT), belief in a controlling God provides a palliative mechanism to cope with a lack of control (Kay et al., 2010). This theory is supported by a large amount of experimental findings showing that inducing a control threat manipulation (e.g., thinking back about a situation in which they lacked control) increased a compensatory efforts for restoring one's sense of control, such as an increased tendency to see illusory patterns (Whitson and Galinsky, 2008) and a preference for stage compared to continuous theories of development and evolution (Rutjens et al., 2013). However, in a registered report study (Hoogeveen et al., 2018) we failed to find evidence for an effect of lack of control on increased belief in a controlling God. However, we found—again in line with CCT—that in the US (but not in the Netherlands), experiencing less control in one's life in general, was associated with an increased belief in a controlling God.

### Health

A wealth of studies have shown the positive effects of believing in God, religious practices (e.g., prayer and church attendance) and religious experiences on feelings of control, mental health and wellbeing (see for instance: Braam and Koenig, 2019; Garssen et al., 2021). However, most of these studies have been conducted in highly religious countries, thereby calling into question the cross-cultural generalizability of these findings. In a large-scale cross-cultural study, involving data collected in 24 countries across 5 continents, we set out to determine the boundary conditions of the religion-health relationship (Hoogeveen et al., 2022b). We used a many-analyst approach, whereby the data analysis was outsourced to 120 analysis teams who independently analyzed the data. Synthesizing the findings from these teams provided strong evidence for the hypothesis that (1) religiosity is indeed positively associated with increased mental and physical wellbeing and (2) that this relationship depends on the perceived cultural norms of religiosity. Specifically: in highly religious countries such as the US or India, being religious is beneficial for one's health, whereas in more secular countries this relationship is absent or even reversed.

In sum, religiosity appears to have a positive relationship with mental health and can provide a sense of control, but only in countries in which being religious is the social norm.

## Socio-cultural mechanisms underlying supernatural beliefs

Cultural-evolutionary accounts of religiosity have pointed out the relevance of socio-cultural factors for the learning and proliferation of supernatural beliefs (Norenzayan and Gervais, 2013), including religious role models and source heuristics.

### Credibility-enhancing displays

CREDS are ostensible markers of religious commitments such as visiting religious services, wearing religious clothing, or adhering to a specific diet. CREDS have been suggested to be a strong predictor of the extent to which supernatural beliefs are transmitted from parents to children, as important role models do not just “talk the talk, but also walk the walk” (Henrich, 2009). This finding fits in a broader literature proposing that ultimately supernatural beliefs subserve an adaptive function by fostering in-group cohesion, cooperation and prosocial behavior (Norenzayan et al., 2016). Indeed, in a cross-cultural study we found that CREDS displayed by one's parents, were the strongest predictor of supernatural beliefs—much more so compared to thinking style, agency detection or mentalizing abilities (Maij et al., 2017). Thus, central role models during one's development, have a strong impact on the proliferation of supernatural beliefs.

### Source heuristics

Next to CREDs, in general people appear more willing to trust information from sources that they credit with authority. The so-called Guru-effect refers to the observation that incomprehensible statements originating from a Guru are perceived to be meaningful, thereby only adding to the status of the Guru (Sperber, 2010). By using a vignette study in which participants were presented with seemingly profound statements that were attributed to a Guru or to a scientist, we found what we dubbed the *Einstein-effect*: across the globe participants rated the statement from the scientist as more profound than from the Guru (Hoogeveen et al., 2022a). We also found that this effect interacted with one's worldview: the Einstein-effect was most pronounced for atheist participants, but religious participants tended to attribute significance to statements from both the scientist and the guru (Hoogeveen et al., 2022a; van der Miesen et al., 2022). Source heuristics provide a proximate mechanism underlying the transmission of supernatural beliefs, and through their down-stream effects on cognitive processing (i.e., the down-regulation of executive functioning; Schjoedt et al., 2011) they also directly underlie the induction of placebo- and expectancy effects.

In sum, we found strong evidence for the role of CREDs and source heuristic effects in the proliferation and acceptance of supernatural beliefs.

## Discussion

Why do some people believe in supernatural phenomena, whereas others don't? The research reviewed in this opinion paper points to the central relevance of socio-cultural factors for acquiring and maintaining supernatural beliefs. Rather than being rooted in deeply engrained tendencies for agency detection, mentalizing, reduced conflict detection or dualistic reasoning, the available evidence points toward the role of cultural scaffolding and explicit teaching for endorsing supernatural beliefs. Children are more likely to endorse the faith of their parents in case their parents engaged in ostensible religious displays. And in general people appear more willing to attribute significance to information from a source they deem trustworthy. Once these supernatural beliefs have been acquired, they encourage a self-sustaining loop by fostering agency-detection experiences, dualistic thinking, and encouraging a more intuitive processing style, providing a feeling of control and even having a protective effect on one's mental and physical health.

## Author contributions

The author confirms being the sole contributor of this work and has approved it for publication.

## Funding

This study was funded by Dr. Rüdiger Seitz, *via* the Volkswagen Foundation, Siemens Healthineers, and the Betz Foundation.

## Conflict of interest

The author declares that the research was conducted in the absence of any commercial or financial relationships that could be construed as a potential conflict of interest.

## Publisher's note

All claims expressed in this article are solely those of the authors and do not necessarily represent those of their affiliated organizations, or those of the publisher, the editors and the reviewers. Any product that may be evaluated in this article, or claim that may be made by its manufacturer, is not guaranteed or endorsed by the publisher.
